# Colorimetric Detection of Dengue by Single Tube Reverse-Transcription-Loop-Mediated Isothermal Amplification

**DOI:** 10.1371/journal.pone.0138694

**Published:** 2015-09-18

**Authors:** Yee-Ling Lau, Meng-Yee Lai, Boon-Teong Teoh, Juraina Abd-Jamil, Jefree Johari, Sing-Sin Sam, Kim-Kee Tan, Sazaly AbuBakar

**Affiliations:** 1 Tropical Infectious Diseases Research and Education Centre (TIDREC), Department of Parasitology, Faculty of Medicine, University of Malaya, 50603, Kuala Lumpur, Malaysia; 2 Tropical Infectious Diseases Research and Education Center (TIDREC), Department of Medical Microbiology, Faculty of Medicine, University of Malaya, 50603, Kuala Lumpur, Malaysia; University of California Davis, UNITED STATES

## Abstract

Dengue is usually diagnosed by isolation of the virus, serology or molecular diagnostic methods. Several commercial kits for the diagnosis of dengue are existing, but concerns have arisen regarding to the affordability and performance characteristics of these kits. Hence, the loop-mediated isothermal amplification (LAMP) is potentially ideal to be used especially in resource limited environments. Serum was collected from healthy donors and patients diagnosed with dengue infection. RNA extracted from the serum samples were tested by reverse-transcription-LAMP assay developed based on 3′-NCR gene sequences for DENV 1–4. Results were interpreted by a turbidity meter in real time or visually at the end of the assay. Sensitivity and specificity of RT-LAMP results were calculated and compared to qRT-PCR and ELISA. RT-LAMP is highly sensitive with the detection limit of 10 RNA copies for all serotypes. Dengue virus RNA was detected in all positive samples using RT-LAMP and none of the negative samples within 30–45 minutes. With continuing efforts in the optimization of this assay, RT-LAMP may provide a simple and reliable test for detecting DENV in areas where dengue is prevalent.

## Introduction

The dramatic global spread and increased frequency and magnitude of epidemic dengue/dengue hemorrhagic fever (DEN/DHF) over the past 40 years underscores the critical need for more effective surveillance, prevention, and control measures. Most countries where DEN/DHF is endemic do not conduct adequate surveillance as a means of assessing disease burden, nor do they possess adequate mosquito control or vaccine prevention programs. The lack of available, affordable, sensitive, and specific diagnostic tests represents the primary hurdle affecting DEN/DHF surveillance in resource limited countries. Early diagnosis of dengue patients is also critical to patient management since it prevents the administration of expensive (and ineffective) antibiotics, expedites the triage of febrile patients to appropriate clinics, and reduces health care costs. With the prospect of specific antiviral therapy in the near future, early diagnosis will be crucial in the identification of patients with dengue, allowing them to receive the proper care. Prompt diagnosis of index cases would also facilitate vector control activities in the community thereby mitigating further transmission.

Currently available diagnostic tests used for dengue diagnosis in most dengue-endemic countries include commercially available IgM- and IgG-based ELISAs. These assays use dengue-specific antigens from all 4 serotypes (DENV 1–4) and are designed to capture anti-dengue specific antibodies present in serum. However, serology tests are usually inconsistent since detection of antibodies requires appropriate collection times and test results may be confused with false-positive reactions due to the presence of antibodies from previous infections.

Virus isolation from acute-phase serum or plasma samples cultured in the presence of the C6/36 mosquito cell line remains the "gold standard", although the disadvantage of this approach is that results are not available for >7 days. Furthermore, isolation of dengue virus from clinical samples using the cell culture approach has generally been unsuccessful due to the fastidious nature of the virus and the low level of transient viremia associated with the disease process [1].

Due to the ability of molecular techniques to provide diagnostic information rapidly, reverse transcription (RT)-PCR, nested PCR, nucleic acid sequence-based amplification (NASBA), and real-time PCR are used to diagnose dengue virus serotypes. The requirement to maintain reagents in cold and the need of expensive thermal cyclers (followed by gel electrophoresis) limits the utilization of these approaches [2]. However, the simplicity and high efficiency of LAMP to rapidly amplify DNA under isothermal conditions suggested that LAMP could be a potential alternative for detecting dengue virus especially in field settings as compared to qRT-PCR which is time-consuming and require RT-PCR machine.

Loop-mediated isothermal amplification (LAMP) originally developed by Notomi *et al*. (2000) represents a very sensitive, easy to use, and less time consuming diagnostic method [3]. LAMP can amplify up to 10^9^ copies in less than1 h under isothermal conditions (65°C) using simple incubators such as water baths or heating blocks making this approach suitable for field work. The method uses 4–6 primers that recognise 6–8 distinct regions of the target DNA which helps eliminate nonspecific binding and increases the specificity of this assay [3].

Since LAMP does not require any major equipment, it represents an ideal diagnostic tool for use in areas with limited resources. A LAMP-based assay that requires the use of 4 reaction tubes for the detection of dengue virus was developed in 2005 by Parida *et al*. and RT-LAMP (Reverse transcription LAMP) was shown to be highly sensitive; however, it was tested on only 25 positive dengue samples and the specificity was only 93% [4]. To be cost effective, we developed a multiplex RT-LAMP method to detect dengue virus infections using a single tube. In addition, we used hydroxynaphthol blue (HNB) dye for the colorimetric detection of the loop-mediated isothermal amplification reaction. One of the most attractive features of this RT-LAMP assay is that the HNB dye allows the test to be interpreted using the naked eye. This approach has been shown to be a sensitive and simple means of rapidly diagnosing turkey coronavirus infections either directly from feces or in association with virus isolation methods [5].

## Materials and Methods

### Human patient serum samples

The study obtained ethics approval from the UMMC Medical Ethics Committee (Ethics Committee/IRB Reference Number: 908.11). A total of 189 serum samples from patients cl inically suspected with DENV infection were obtained from University of Malaya Medical Centre (UMMC), Kuala Lumpur. All clinical samples were taken as part of standard patient care. The samples were divided in the laboratory to be used for routine diagnosis by serological assays (SD Dengue IgM Capture and SD Dengue IgG Capture ELISA kits, Standard Diagnosis Inc., Korea) and evaluation of the RT-LAMP assay developed in this study. The viral RNA was confirmed by virus culture and *genesig* Real-Time qRT-PCR DENV Detection Kit (PrimerDesign Ltd., UK). As this is a retrospective study, patient samples were included in the study without any specific reference to the patient's identity. Samples collected from healthy donors (n = 24) were from Department of Parasitology, Faculty of Medicine, University of Malaya. All volunteer healthy donors were duly informed before blood was taken and written consent was obtained before the healthy donor samples were used in this study.

### RNA extraction

The genomic viral RNA was extracted from 140 μl of patient serum samples by using the QIAamp viral RNA mini kit (QIAGEN, Hilden, Germany). The RNA was eluted from the QIAspin columns in a final volume of 60 μl of elution buffer and was stored at -70°C until it was used.

### RT-LAMP assay

The serotype-specific oligonucleotide primers used for RT-LAMP assay amplification of dengue viruses were designed using the Primer-Explorer V3 software based on 3′ noncoding region (NCR) (GenBank accession number: DENV-1 Western Pacific, U88535; DENV-2 New Guinea C, AF038403; DENV-3 H87, M93130, and DEN-4 China Guangzhou B5, AF289029) (Table 1) [6]. Loop primers (Loop-F and Loop-B) were designed manually. The RT-LAMP assay was performed using Loopamp RNA amplification kit (Eiken Chemical Co. Ltd., Japan). Briefly, a 25 μl reaction mixture consisted of 1.6 μM FIP and BIP primer, 0.8 μM Loop-F and Loop-B primer, 0.2 μM F3 and B3 primer, 12.5 μl of 2X reaction mixture (RM) (provided in the kit), 1 μl of Enzyme mixture (EM) (provided in the kit), 0.7 μl of sterile deionize water, 1μl of Florescent Detection reagent (FD) (provided in the kit) and 1 μl of template RNA. Single tube RT-LAMP was conducted by adding all primer sets in a single reaction mixture. The reaction mixture was carried out in a Loopamp real-time turbidimeter (LA-320, Teramecs, Co., Ltd., Japan). To prevent cross-contamination, different sets of pipettes and different work areas were designated for template preparation, reaction mixture preparation and DNA amplification. All experiments were performed in duplicate at least 2 times.

**Table 1:** RT-LAMP Primers used in this study.

Virus Serotype	Primer	Sequence (5'→3')
DENV-1	F3	TGGGGTAGCAGACTAGTGG
	B3	TCTGTGCCTGGAATGATGC
	FIP	CCACCAGGGTACAGCTTCCCGACCCCTCCCAAAACACAA
	BIP	AGAGGTTAGAGGAGACCCCCC-AGCAGGATCTCTGGTCTCTC
	FLP	TGGTGTTGGGCCCCGCT
	BLP	ACAGCATATTGACGCTG
DENV-2	F3	TACGCATGGCGTAGTGGA
	B3	GCGTTCTGTGCCTGGAAT
	FIP	TCATCTCACCTTGGGCCCCCCGGTTAGAGGAGACCCCTC
	BIP	AGAGGTTAGAGGAGACCCCCCGCAGGATCTCTGGTCTTTCC
	FLP	GTTGCTGCGATTTGTAA
	BLP	ACAGCATATTGACGCTG
DENV-3	F3	GCTGTACGCACGGTGTAG
	B3	CCTGGAATGATGCTGAGGAG
	FIP	GGTACAGCTTCCCTCAGTGCTCGTGGTTAGAGGAGACCCCT
	BIP	AGAGGTTAGAGGAGACCCCCCAGCAGGATCTCTGGTCTCTC
	FLP	CTGCTGCGTTGTGTCAT
	BLP	CAGCATATTGACGCTGG
DENV-4	F3	CGCGTGGCATATTGGACTA
	B3	TGCCTGGATTGATGTTGTA
	FIP	CAGCTTCCTCCTGGCTTCGGCGGTTAGAGGAGACCCCTC
	BIP	AGAGGTTAGAGGAGACCCCCCAGGATCTCTGGTCTTTCCCA
	FLP	CCCCTTTTGCTGCGTTT
	BLP	AAACAGCATATTGACGC

### Real-time monitoring of amplification by the RT-LAMP assay

The real-time monitoring of amplification of the dengue RT-LAMP assay was observed with Loopamp real-time turbidimeter (LA-320, Teramecs, Co., Ltd., Japan). The reaction is considered positive when the turbidity reached 0.1 within 60 min at 650 nm. The time needed for the turbidity of each tested sample to exceed at 0.1 is referred to as the threshold time (Tt) [7].

### Endpoint assessment

HNB dye (Sigma, USA) was dissolved in distilled water at 20 mM to prepare a stock solution. The LAMP assay containing 120 μM of HNB dye was performed in a 25 μl reaction mixture with the same components as in the RT-LAMP assay except excluding the (FD) reagent. Turbidity of the RT-LAMP assay was observed with the naked eye. A positive reaction was indicated by a color change from violet to sky blue. On top of that, PCR was performed on 4 μL LAMP products using F3 and B3 primers with the same reaction mixture as above with the following PCR cycles: 94°C for 4 min; 35 cycles at 94°C for 30 s, annealing at 55°C for 1 min, extension at 72°C for 30 s.

### Positive control plasmid DNA

For sensitivity assessment, plasmids containing the target region of the 3′-NCR gene were constructed for each serotype for use in the LAMP reaction. The target DNA sequence was amplified with 2 LAMP primers (F3 and B3 primers) by PCR and was then cloned into the pGEM-T cloning vector (Promega, USA). Plasmid DNA purification was performed with a QIAprep Miniprep kit (QIAGEN, Hilden, Germany). The resulting sequences were aligned using the 3′-NCR gene sequences for DENV1-4 in GenBank to confirm that the target sequences were correct.

### Analytical sensitivity of LAMP primers

Positive control plasmid DNAs were used to determine the minimum copy number (lower detection limit) of the target gene sequence detectable by LAMP. The standard curve for LAMP was constructed using 10-fold serial dilutions of plasmid DNA (106 copies to 1 copy) to sterile water. DENV RNA was extracted from the culture supernatant DENV-1 genotype I, II, III; DENV-2, Asian I and cosmopolitan; DENV-3, genotype I, II and III and DENV-4, sub genotype IIa and IIb) [8,9,10,11]. The sensitivity of RT-LAMP assay was assessed using a panel of serially diluted viral RNA (1000, 100, 10, and 1 copy numbers) extracted from culture supernatant. The viral RNA used was quantitated using the genesig Real-Time qRT-PCR DENV Detection Kit (PrimerDesign Ltd., UK) as previously described. The sensitivity test of LAMP primers was performed in duplicate and repeated 2 times.

### Analytical specificity of LAMP primers

Specificity of the LAMP primers were tested against DENV-1 genotype I, II, III; DENV-2, Asian I and cosmopolitan; DENV-3, genotype I, II and III and DENV-4, sub genotype IIa and IIb) [8,9,10,11]. All the viruses were archived in the University Malaya Medical Centre (UMMC) Diagnostic Virology Laboratory repository and 3 closely related arboviruses common in the region; Japanese encephalitis virus (JEV), Chikungunya virus (CHIKV), and Sindbis virus (SINV) with the same condition as mentioned above. The specificity test for LAMP primers was performed in duplicate and repeated 2 times.

### Clinical sensitivity and specificity

The clinical sensitivity and specificity of RT-LAMP assay was calculated based on the screening of the 189 suspected dengue and 24 healthy donor serum samples. Sensitivity was calculated as (number of true positives)/(number of true positives + number of false negatives), and specificity was calculated as (number of true negatives)/(number of true negatives + number of false positives). Positive predictive value (PPV) and Negative predictive value (NPV) were calculated based on an average of 84% of dengue prevalence in Malaysia [12]. PPV = (sensitivity X prevalence)/ [sensitivity X prevalence + (1-specificity) X (1-prevalence)], NPV = [specificity X (1-prevalence)]/ [(1-sensitivity) X prevalence + specificity X (1-prevalence)]. A composite diagnosis for each sample (2 out of 3 tests based on qRT-PCR, ELISA and RT-LAMP giving the same result) was used as a reference.

## Results

### Optimization of LAMP assay

**Fig 1:**
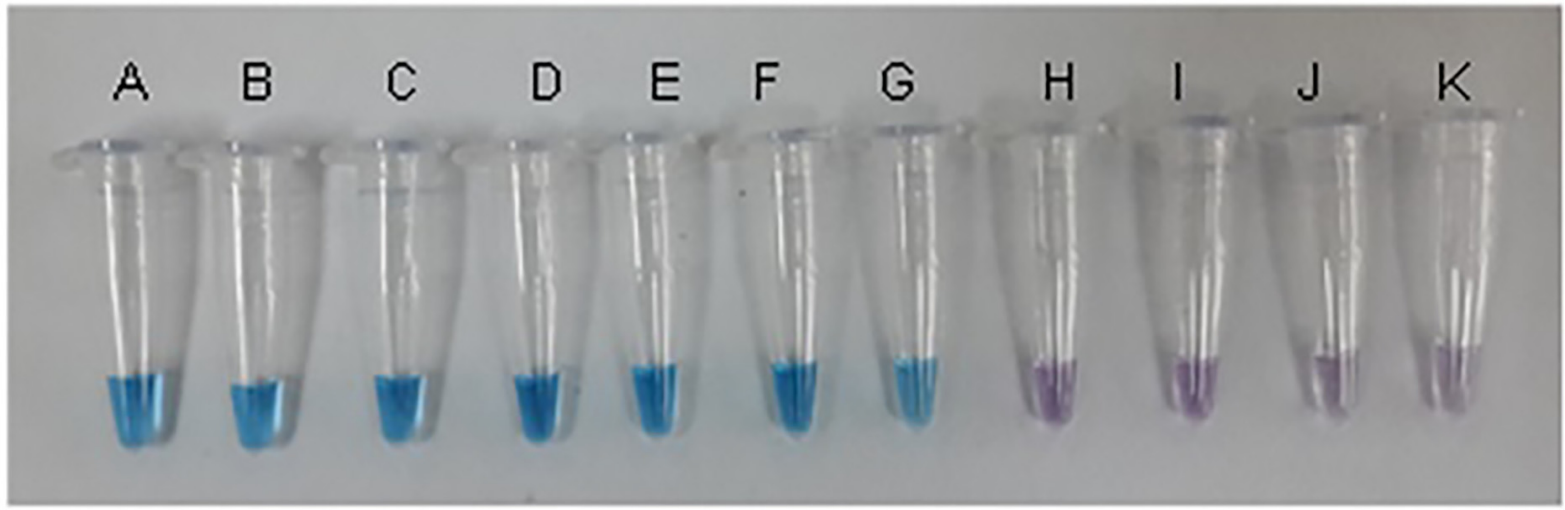
Visualization of LAMP assay products following addition of HNB dye. The color changes from violet (negative reaction) to sky blue (positive reaction). Tube A and B: DENV 1 serotypes; tube C and D: DENV 2 serotypes; tube E and F: DENV 3 serotypes; tube G: DENV 4 serotype; tube H: JEV; tube I: CHIKV; tube J: SINV; tube K: Negative control (distilled water).

**Fig 2:**
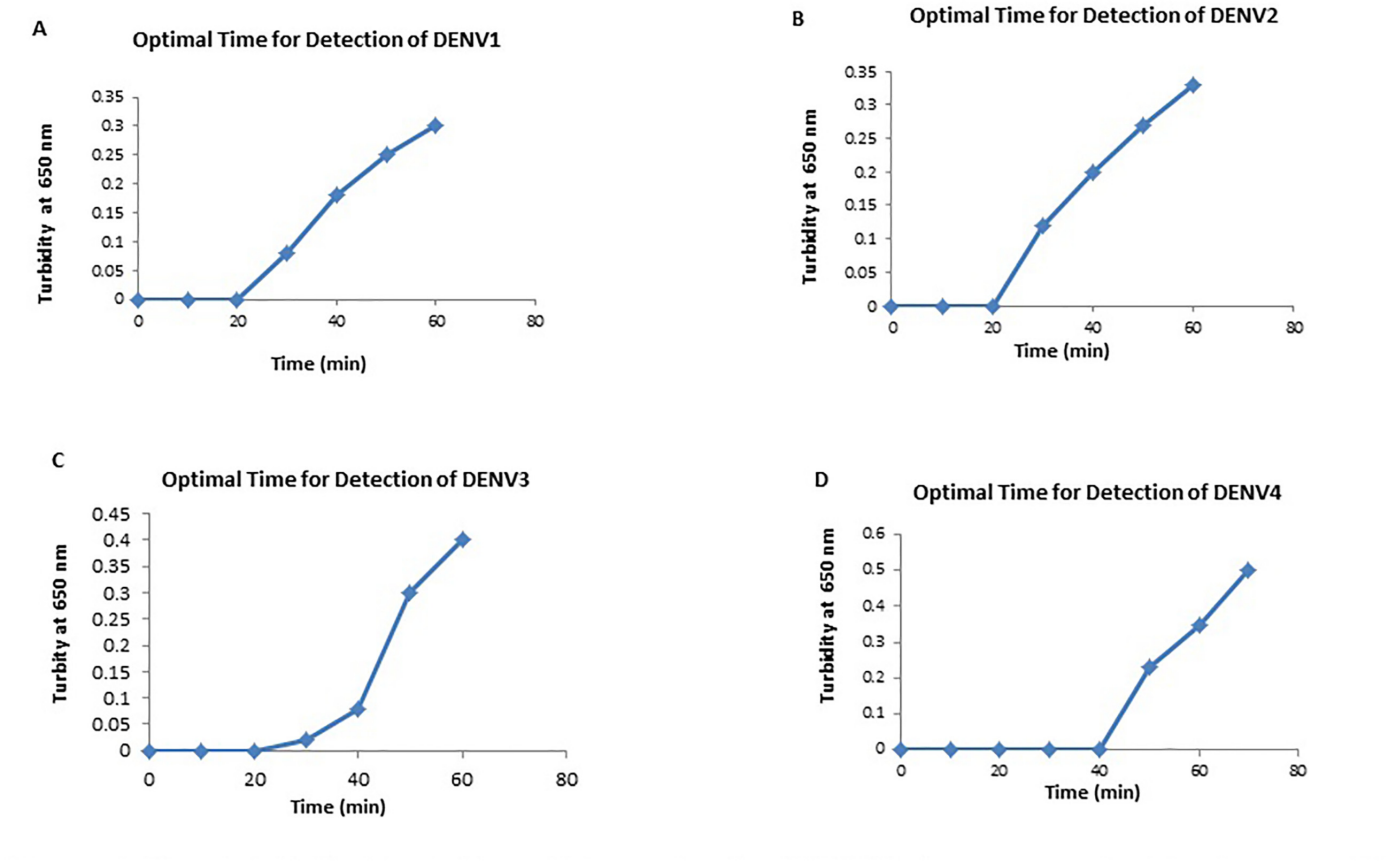
Optimal time of detection for DENV 1, DENV 2, DENV 3 and DENV 4 serotypes. Panel A-C: optimal time of detection for DENV 1–3 serotypes is 30 min; panel D: optimal time of detection for DENV 4 serotype is 45 min.

By using the primer sets selected, a single-tube one-step RT-LAMP assay system was standardized for rapid detection of all four serotypes of dengue viruses. The LAMP reaction was performed using plasmid DNA as template to determine the optimal reaction conditions. The amplification was observed as a typical ladder of multiple bands on the agarose gel. The monitoring of RT-LAMP assay amplification was carried out by naked-eye inspection following addition of HNB dye and real-time turbidimeter. Positive reaction was showed by a color change from violet to sky blue (Fig 1). Temperature of 65°C was found to be optimal and the time of positivity observed through naked-eye and real-time monitoring using Loopamp real-time turbidimeter was found to be 30 min for DENV 1, 2 and 3 serotypes and 45 min for DENV 4 serotype (Fig 2). The positive color (sky blue) and turbidity was only observed with the reference virus, whereas none of the control viruses showed a color change.

### Specificity of LAMP primers

Various RNAs comprising of DENV1-4, healthy donors, Japanese encephalitis virus (JEV), Chikungunya virus (CHIKV), and Sindbis virus (SINV) were used as template in the RT-LAMP assay to investigate the specificity of LAMP primers. All dengue positive samples showed positive results while other viruses and healthy donor samples were all negative under RT-LAMP.

### Sensitivity of RT-LAMP detection

The detection limit of RT-LAMP for 3’-NCR was as low as ten copies. A total of 115 out of 189 samples were detected positive under RT-LAMP. ELISAs and qRT-PCR detected 162 and 98 positive samples respectively (Table 2). Clinical sensitivity of RT-LAMP, qRT-PCR and ELISA was 100%, 85.2%, and 100% respectively (Table 3).

**Table 2:** Total dengue samples detected by ELISA, qRT-PCR and RT-LAMP.

Method	Positive	Negative	Total
ELISA	162	51	213
qRT-PCR	98	115	213
RT-LAMP	115	98	213
Reference*	115	98	213

Note:

*A composite diagnosis for each sample based on two out of three methods giving the same result was used as reference. RT-LAMP = Real-time loop-mediated isothermal amplification; qRT-PCR = Quantitative Real-Time polymerase chain reaction.

**Table 3:** Percentage of Sensitivity, specificity, PPV and NPV of ELISA, qRT-PCR and RT-LAMP for detection of dengue virus infection in human samples.

Method	%Sensitivity(95%CI)	%Specificity(95%CI)	%PPV(95%CI)	%NPV(95%CI)
ELISA	100 (96.8–100)	52 (41.7–62.2)	91.6 (87.33–95.9)	100
qRT-PCR	85.2 (77.4–91.2)	100 (96.3–100)	100	55.9 (46.8–65.0)
RT-LAMP	100	100	100	100

Note: PPV = Positive predictive value; NPV = Negative predictive value.

## Discussion

Loop-mediated isothermal amplification (LAMP) represents a novel DNA amplification method developed by Notomi et al. (2000) that within 1 h (at 65°C) effectively provided diagnostic results [3]. LAMP is easy to use, convenient, and cost-effective, requiring minimal equipment such as water baths or heating blocks. Therefore, it has been widely applied as a diagnostic tool for several viral, bacterial, and parasitic diseases [13–15]. Parida et al. (2005) reported a one-step RT-LAMP for the detection of 4 dengue serotypes (in separate tubes) by targeting the 3’-NCR [4]. In 2011, Li et al. developed a single tube reaction system for the detection of DENV infection using RT-LAMP primers based on amplification of the C-prM gene; however, this gene is relatively less well conserved among all 4 DENV serotypes (inter-serotype) [16]. These 2 studies evaluated their RT-LAMP assays based on their ability to detect DENV infections using a small clinical sample size (<70). In 2013, Teoh et al. reported a single-tube reverse RT-LAMP assay targeting the 3’ untranslated region (UTR) for the detection of all 4 DENV serotypes in 171 confirmed dengue samples. However the sensitivity of this RT-LAMP assay was only 92.5% compared to qRT-PCR [17]. In the present study, an one step RT-LAMP assay was designed to amplify the 3’NCR capable of diagnosing the 4 dengue serotypes. In addition to the real-time monitoring provided by the present LAMP test, this study was the first to apply HNB dye for the detection of amplified targets using the naked eye following 30–45 min amplification step at 65°C. The amplification time for our RT-LAMP assay (based on the 3’NCR) was significantly shorter compared to previously developed RT-LAMP assays targeting the 3’UTR [17].

RT-LAMP assay designed in this study was shown to be 100% sensitive and specific which is better as compared to qRT-PCR (Table 3). The date of illness onset ranged from day 1 to day 11 of illness. DENV viral loads detected on day 1 to day 9 ranged from 2.97 to 6.67 log10 DENV RNA copies/ml of serum. Viral loads of 2.93 log10 RNA copies/ml (equivalent to 10 RNA copies) were beyond the detection limit of qRT-PCR [18]. This is one of the possible reasons of false-negative qRT-PCR results. The simplicity and high efficiency of LAMP to rapidly amplify DNA under isothermal conditions suggested that LAMP could be a potential alternative for detecting dengue virus especially in field settings as compared to qRT-PCR which is time-consuming and require RT-PCR machine [19–21]. Another advantage of using the LAMP assay is the colorimetric detection of positive reactions that allows positive and negative amplifications to be distinguished based on observed color changes with the naked eye.

## Conclusions

Based on the results obtained in this study, the RT-LAMP assays represents a potential alternative for the molecular diagnosis and routine screening of dengue virus infections, especially in dengue endemic countries. It could also be useful in monitoring the efficacy of dengue control and eradication programs.
